# Low temperature reduction of hexavalent chromium by a microbial enrichment consortium and a novel strain of *Arthrobacter aurescens*

**DOI:** 10.1186/1471-2180-6-5

**Published:** 2006-01-25

**Authors:** Rene' N Horton, William A Apel, Vicki S Thompson, Peter P Sheridan

**Affiliations:** 1 Department of Biological Sciences, Idaho State University, Campus Box 8007, Pocatello, ID USA 83209-8007; 2 Idaho National Laboratory, P.O. Box 1625, Idaho Falls, ID USA 83415

## Abstract

**Background:**

Chromium is a transition metal most commonly found in the environment in its trivalent [Cr(III)] and hexavalent [Cr(VI)] forms. The EPA maximum total chromium contaminant level for drinking water is 0.1 mg/l (0.1 ppm). Many water sources, especially underground sources, are at low temperatures (less than or equal to 15 Centigrade) year round. It is important to evaluate the possibility of microbial remediation of Cr(VI) contamination using microorganisms adapted to these low temperatures (psychrophiles).

**Results:**

Core samples obtained from a Cr(VI) contaminated aquifer at the Hanford facility in Washington were enriched in Vogel Bonner medium at 10 Centigrade with 0, 25, 50, 100, 200, 400 and 1000 mg/l Cr(VI). The extent of Cr(VI) reduction was evaluated using the diphenyl carbazide assay. Resistance to Cr(VI) up to and including 1000 mg/l Cr(VI) was observed in the consortium experiments. Reduction was slow or not observed at and above 100 mg/l Cr(VI) using the enrichment consortium. Average time to complete reduction of Cr(VI) in the 30 and 60 mg/l Cr(VI) cultures of the consortium was 8 and 17 days, respectively at 10 Centigrade. Lyophilized consortium cells did not demonstrate adsorption of Cr(VI) over a 24 hour period. Successful isolation of a Cr(VI) reducing organism (designated P4) from the consortium was confirmed by 16S rDNA amplification and sequencing. Average time to complete reduction of Cr(VI) at 10 Centigrade in the 25 and 50 mg/l Cr(VI) cultures of the isolate P4 was 3 and 5 days, respectively. The 16S rDNA sequence from isolate P4 identified this organism as a strain of *Arthrobacter aurescens*, a species that has not previously been shown to be capable of low temperature Cr(VI) reduction.

**Conclusion:**

*A. aurescens*, indigenous to the subsurface, has the potential to be a predominant metal reducer in enhanced, *in situ *subsurface bioremediation efforts involving Cr(VI) and possibly other heavy metals and radionuclides.

## Background

Chromium is a transition metal most commonly found in the environment in its trivalent (Cr^3+^) and hexavalent (Cr^6+^) forms [1]. Naturally occurring Cr is almost exclusively in the trivalent state, as the energy required for its oxidation is high. Hence, the hexavalent form is usually considered to be a man-made product [2]. The toxicities of the two forms of chromium are vastly different. Trivalent chromium is generally a nontoxic, nonmobile micronutrient [3]. Hexavalent chromium is water soluble, toxic, and carcinogenic, and is considered a pollutant by the United States Environmental Protection Agency (EPA) [4]. Chromium is the second most common inorganic contaminant of groundwater at hazardous waste sites [5]. The solubility and negative charge of its more common forms, chromate and dichromate (CrO_4_^2-^, and HCrO_4_^-^), lead to limited adsorption in aquifer minerals, and results in high mobility of Cr^6+ ^in aquifers [6]. The historical and present day contamination of groundwater and soils by Cr^6+ ^is a result of its industrial uses, including metal plating (for corrosion resistance), pigment production, and lumber and wood products (for preservation) [7].

Many water sources are at low temperatures year round (≤15°C) and it is important to evaluate the possibility of remediating Cr^6+ ^contamination using microorganisms adapted to these low temperatures (psychrophiles). Limitations of bioremediation processes at low temperatures have been described in the past as having slow biomass build-up rates, slow degradation and low loads [8]. Furthermore, many bioremediation processes depend on anaerobic Cr^6+ ^reduction and it is commonly believed that anaerobic bioreactors are particularly hard to operate at ambient groundwater temperatures [8]. Recent efforts have tested the possibilities for aerobic and anaerobic low temperature bioremediation of contaminants other than Cr^6+ ^including biostimulation and bioaugmentation [9-11].

To date, there have been few reports of psychrophilic Cr^6+^-reducing organisms [12]. Mesophilic genera capable of Cr^6+ ^reduction include: *Acinetobacter *[13], *Aerococcus *[14], *Aeromonas *[14], *Aspergillus *[15], *Bacillus *[16], *Corynebacterium *[17], *Deinococcus *[18], *Desulfomicrobium *[19], *Desulfovibrio *[20], *Enterobacter *[21-23], *Escherichia *[24,25], *Microbacterium *[26], *Micrococcus *[14], *Ochrobactrum *[13], *Pseudomonas *[27-29], *Rhodobacter *[30], *Shewanella *[31], *Staphylococcus *[32], *Streptomyces *[33], *Vibrio *[34], and *Zoogloea *[35]. Since mesophilic Cr^6+ ^reduction can proceed both aerobically and anaerobically [36], it is reasonable to assume that psychrophilic reductions will also proceed both aerobically and anaerobically. Most studies referenced were performed at temperatures at or above 20°C. The single low temperature (10°C) study involving a soil community and varying electron acceptors yielded significant reduction of Cr^6+ ^[12]. To the best of our knowledge, no low temperature groundwater studies (saturated zone of aquifer) on the reduction of Cr^6+ ^have been performed. The use of indigenous psychrophilic microorganisms may provide insight into many of the problems associated with low temperature remediation.

This study used samples obtained from a Cr^6+ ^(~1.3 mg/l) contaminated site within the Hanford aquifer  as inocula from which indigenous psychrophilic microorganisms were cultured and tested for their ability to reduce Cr^6+ ^to levels below the required EPA minimum. Identification of psychrophilic Cr-reducing community members will allow future studies of remediation possibilities using indigenous populations at other sites as well as help guide the search for other closely related psychrophilic microorganisms for use in remediating Cr^6+ ^present in low temperature environments.

## Results

### Enrichments

Enrichments with Cr^6+ ^concentrations from 0 to 400 mg/l showed growth in the form of turbidity (cell density approximately 10^8^/mL) at 10°C. Subsequent transfers of cultures to like concentrations of Cr^6+^-containing media also produced turbidity. Clearing of the VB broth (addition of chromate turned the broth yellow) to colorless or pale white and formation of a white precipitate occurred in the 30 and 60 mg/l Cr^6+ ^concentration enrichments and in a preliminary reduction study, but not in the uninoculated controls. Enrichments attempted in media containing Cr^6+ ^concentrations of >1000 mg/L grew poorly.

### Consortium reduction experiments

**Figure 1:**
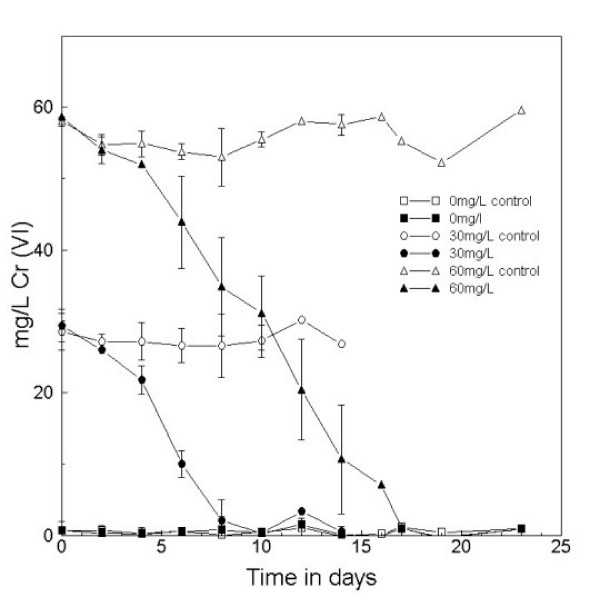
**Cr^6+ ^reduction by enrichment consortia under aerobic conditions at 10°C**. Upon complete reduction of Cr^6+ ^no further data was collected and is represented on the graph by the truncation of the lines for both the reduction and its similar control.

All enrichment cultures showed evidence of Cr^6+ ^removal as the decolorization of media (from yellow to clear) and measurement of the decreasing Cr^6+ ^concentration by the diphenyl carbazide assay. Standards and controls in each of the experiments were used to compare the amount of Cr^6+ ^left in the media at approximately 48 hour intervals. No reduction was observed in cell-free controls. The overall averages and standard deviations of the three samples taken from each of the triplicate tubes in the three serial reduction experiments are represented by data provided in Figure 1. The enrichments containing 30 mg/l Cr^6+ ^were completely reduced in approximately 8 days at 10°C and the 60 mg/l Cr^6+ ^proceeded to zero in less than 17 days at 10°C.

### Isolations

In order to select for organisms that were both resistant to Cr^6+ ^and able to reduce Cr^6+^, the original enrichments were performed at high levels of Cr^6+^. Streaking for isolation on VB plates (without Cr^6+^) from a 1000 mg/l Cr^6+ ^liquid enrichment yielded an isolate (designated P4).

### Isolate reduction experiments

**Figure 2:**
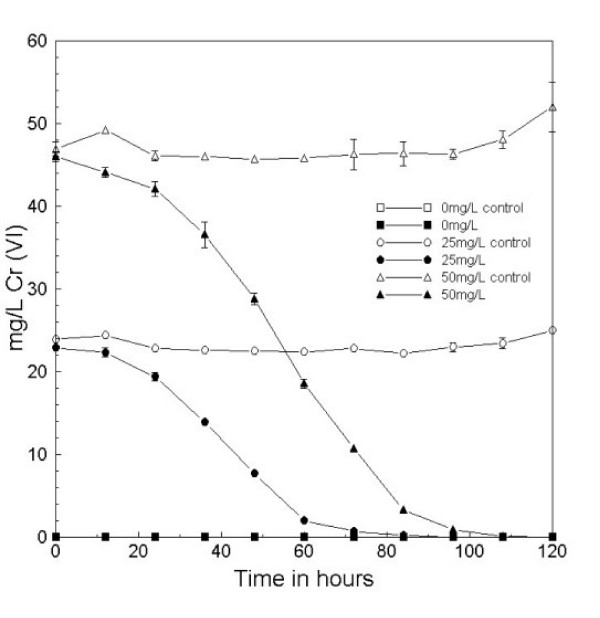
Cr^6+ ^reduction by isolate P4 under aerobic conditions at 10°C.

Isolate P4 cultures showed evidence of Cr^6+ ^removal in VB media as the decolorization of media (from yellow to clear) and measurement of the decreasing Cr^6+ ^concentration by the diphenyl carbazide assay similar to the consortium reduction experiments. Standards and controls in each of the experiments were used to compare the amount of Cr^6+ ^left in the media at approximately 12 hour intervals. P4 grew poorly in VB media without the addition of Cr^6+^. No reduction was observed in the cell-free controls. The overall averages and standard deviations of the three samples, taken from each of the triplicate tubes in the three serial reduction experiments, are represented by data provided in Figure 2. The enrichments containing 25 mg/l Cr^6+ ^were completely reduced in less than 72 hours at 10°C and the 50 mg/l Cr^6+ ^proceeded to zero in less than 120 hours at 10°C.

### Consortium adsorption experiments

Cr^6+ ^removal was not evidenced in adsorption experiments. Consortium cells suspended in deionized water and Cr^6+ ^retained the yellow color of Cr^6+^-contaminated media. Concentrations of Cr^6+ ^were statistically unchanged by the end of the 24 hour period as measured using the diphenyl carbazide assay. Temperature did not affect the adsorption experiments.

### 16S rDNA Identification and phylogenetic analysis of isolate P4

**Figure 3:**
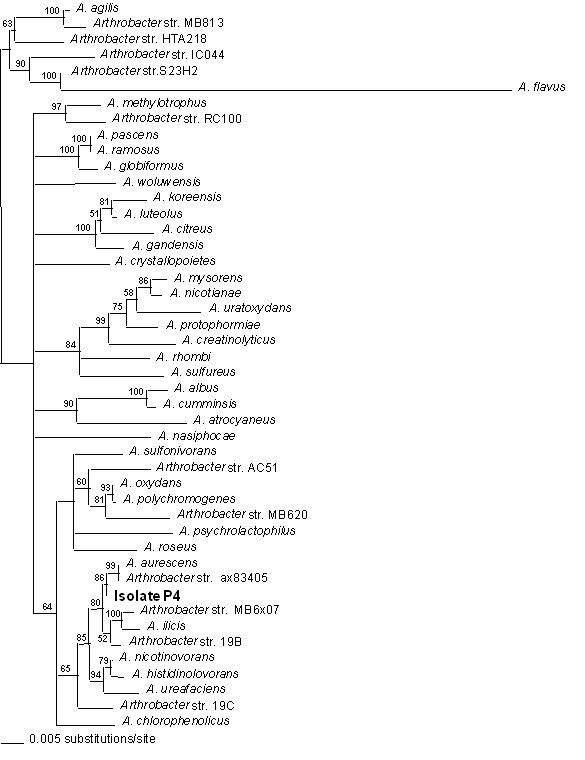
Distance Analysis phylogenetic tree of *Arthobacter *species including the isolate P4.

PCR amplification and subsequent sequencing yielded an approximately 1.5 kbp DNA fragment consistent with the expected length of the amplification product. A fragment of 1373 unambiguous bases was used in the search and analysis of related microorganisms. Ribosomal Database Project and Genbank database searches both resulted in the high sequence homology (1369/1373 bases) to *Arthrobacter aurescens*. Subsequent phylogenetic analysis also revealed the isolate to be a strain of *A. aurescens *(Figure 3).

## Discussion

This study demonstrates that indigenous microbial populations present in Cr^6+^-contaminated aquifers are able to aerobically catalyze the removal of toxic and soluble Cr^6+ ^from the media, most likely reducing it to the relatively nontoxic and insoluble Cr^3+^. The absence of Cr^6+ ^in the media, in addition to the lack of adsorption demonstrated by the three separate adsorption experiments, suggests the Cr^6+ ^was reduced to the less toxic Cr^3+ ^form. Further experimentation with cell lysates of P4 at 18°C showed Cr^6+^-reduction activity in the soluble protein fraction, not the membrane bound protein fraction, also suggesting enzymatic reduction (data not shown). The low temperature (10°C) used in the experiments and the timeline for the reductions also suggests that Cr^6+ ^can be remediated in a reasonable amount of time at the low environmental temperatures present in many aquifers. In comparison, a mesophilic isolate from another study, *Arthrobacter crystallopoites *strain ES 32 [37,38] reported a lower rate of chromate reduction at 30°C when compared to isolate P4 at 10°C. Interestingly, ES 32 had a higher temperature optimum for Cr^6+ ^reduction than for optimal cell growth [38]. The lack of previous low temperature studies is clearly demonstrated by a search of the literature in which only a single paper by Tseng and Bielefeldt [12] on the low temperature biotransformation of hexavalent chromium in soil is found.

Both the consortium and P4 isolate cultures were shown to grow and reduce Cr^6+ ^at 10°C. Significant biomass of the P4 isolate could be generated within 2 days of growth in R_2 _broth at 10°C (cell densities of 10^8^/ml). Studies using mesophilic microorganisms from genera such as *Bacillus, Pseudomonas *and *Escherichia *[16,28,39] all required incubation at temperatures well above those used in this study and those found in the aquifer environment we are targeting for bioremediation.

A number of studies suggest both growth-dependent and growth-independent chromium reduction [20,29,40]. In either case, chromium reduction does seem to be biomass dependent in our study as well as in others [21,41]. The lag at the beginning of the consortium reduction experiments as well as observations of increased turbidity throughout the experiments suggests that adequate cell biomass must be produced before reduction begins in earnest. Bopp and Ehrlich [28] showed that higher concentrations (1000 mg/l) of Cr^6+ ^produced a much longer lag phase and a significantly lower final yield of biomass than lower concentrations. The reduced biomass would also contribute to the lack of complete reduction found at higher concentrations in many studies [22,25] as well as in the higher concentrations tested in our lab (data not shown). Previous studies using cellular biomass grown on uncontaminated substrates to test Cr^6+ ^reduction greatly decreased the amount of time required to completely reduce Cr^6+ ^[21,39], similar to our findings with the isolate P4 reduction experiments (Figure 2). Increased turbidity after only 24 hours in R_2 _broth at 10°C (grown aerobically) and the achievement of stationary phase (as determined by absorbance readings, 1:10 dilution in R2 broth, OD = 0.16) after 3 days suggests that P4 is relatively fast growing. P4 grew at 10, 18, and 25°C but not at 37°C suggesting the isolate is a true psychrophile. Growth appeared fastest at 18°C. The ability to increase biomass in a short time given the proper nutrients suggests that P4 could be useful in bioremediation using nutrient addition.

The enrichment culture and isolate P4 consistently reduced Cr^6+ ^in VB medium up to concentrations of 60 mg/l Cr^6+^. Higher concentrations seemed to inhibit reduction, although growth was slower but still observed as turbidity in the enrichments (data not shown). Dilution of the Cr^6+ ^at 1000 mg/L may have affected the limited range of the diphenyl carbazide assay, causing the appearance of the lack of reduction at higher concentrations. Both the consortium and isolate P4 showed significant tolerance of Cr^6+ ^up to concentrations of 1000 mg/l (data not shown) as well as measurable reduction over short periods of time at concentrations up to 60 mg/l Cr^6+^. This tolerance is greater than or comparable to most mesophilic microorganisms tested, such as *Pseudomonas fluorescens *at 53.5 mg/l [27] and *Bacillus sp*. at 500 mg/l [42]. Furthermore, the isolate P4 and consortium reductions presented here occurred at temperatures close to 30°C lower than in the studies using mesophilic organisms, suggesting that the enzyme(s) responsible for the reduction are truly cold-active.

Complete reduction was observed in all experiments (both consortium and isolate P4) with concentrations of Cr^6+ ^up to 60 mg/l (Figures 1&2) suggesting that complete reduction in the environment is also possible. The lack of reduction in the sterile controls along with the lack of Cr^6+ ^adsorption to cell biomass in the three adsorption experiments suggests that the members of the enrichment community (which included isolate P4) were responsible for the reduction of Cr^6+^. Since most aquifers contaminated with Cr^6+ ^have levels below 60 mg/l, these experiments would also suggest remediation of the lower levels of Cr^6+ ^contamination present in aquifers is possible. Bioremediation literature suggests low levels of contamination are very difficult to completely remediate. Lack of induction of enzyme systems at low contaminant concentrations and problems with availability of contaminants bound to organics and sequestered in other matrices all contribute to persistence of contaminants in the environment. It has also been suggested that indigenous microorganisms may be more successful in reducing low contaminant concentrations [8]. The complete reduction of Cr^6+ ^at 10°C in this study using an indigenous member of the Hanford microbial community and past studies with indigenous mesophilic microorganisms suggest that there are environmental candidates for reduction of the low levels of contamination usually found in aquifers [23,43].

Studies have shown *Arthrobacter *species adsorbing Fe, Cd, and Cu, but not Cr [44,45]. Chromium has, however, been shown to adsorb to both *Shewanella *and *Bacillus *species [46]. Adsorption studies performed on the Hanford consortium in our laboratory (which included the isolate P4) did not show significant removal of Cr^6+ ^due to adsorption. Three separate adsorption studies used killed (autoclaved) cells, metabolically inhibited live cells, or lyophilized cells. None of these studies showed significant adsorption of Cr^6+ ^within 24 hours of Cr^6+ ^addition. These, along with studies showing activity in the soluble fraction of lysate, suggest enzymatic reduction.

A few *Arthrobacter *species, like *A. oxydans *and *A. crystallopoites *strain ES32 have been noted previously to reduce Cr^6+ ^[37,47]. Identification of *A. oxydans *to the species level in the previous study was performed via Fatty Acid Methyl Ester (FAME) analysis, while ES 32 was characterized by 16S rDNA sequencing. Carmargo et al. [37] showed ES 32 to have its optimum Cr^6+ ^reduction in a temperature range of 30–35°C, but did not test its Cr^6+ ^reduction rates below 25°C. P4, by comparison, grew well and reduced Cr^6+ ^at 10°C at a faster rate than ES 32 at 30–35°C. Further comparison of the two organisms (P4 and ES 32) reveals a much lower starting concentration of Cr^6+ ^for the reduction studies using ES 32 (1.04 mg/L [38] and 2.0 mg/L [37]). Reduction of Cr^6+ ^using P4 at 10°C proceeded at a rate 5.5 times faster than ES 32 at 30°C (0.72 mg/L/h and 0.13 mg/L/h respectively, calculated using the linear portion of the reduction curve for both organisms). As for Cr^6+ ^resistance, isolate P4 tolerated up to 1000 mg/L Cr^6+ ^while isolate ES 32 was from a group that had low tolerance above 500 mg/L Cr^6+ ^[37]. Within these comparisons, isolate P4 is more resistant and reduces higher concentrations of Cr^6+^at a significantly faster rate.

In the current study, the identification of isolate P4 as a strain of *A. aurescens *was performed via 16S rDNA sequencing with comparison to sequences found in the Ribosomal Database Project and the GenBank database at NCBI. To the best of our knowledge, the species *A. aurescens *has not been previously associated with Cr^6+ ^reduction.

## Conclusion

Considering the ubiquity of organisms in the genus *Arthrobacter*, we suggest further exploration of the *in situ *metal reduction potential of this under-studied genus. Resistance and tolerance of *Arthrobacter spp*. have been demonstrated to a wide variety of heavy metals including mercury, chromium, lead, nickel and copper [48,49]. That, together with *Arthrobacter*'s ability to reduce Cr^6+ ^and other toxic metals, indicates that *Arthrobacter spp*. indigenous to the subsurface have potential to be useful metal reducers in enhanced, *in situ*, subsurface bioremediation efforts involving Cr^6+ ^and other heavy metals and radionuclides.

## Methods

### Sampling, enrichment and isolation

A core from the saturated zone of the Ringold Formation at 25.9 meters below ground surface was obtained from a Cr^6+ ^contaminated area on the U.S. Department of Energy's Hanford facility. The concentration of Cr^6+ ^in the aquifer was 1.49 mg/l. The core (10.2 cm diameter) was collected in a polycarbonate liner and shipped refrigerated in an argon-filled, air-tight paint can. Upon receipt, the core was refrigerated until it was aseptically pared to expose uncontaminated, internal regions that served as inocula for the experiments described below.

A 4.9 g Hanford aquifer core sample was mixed with 10 ml of Vogel Bonner (VB) broth [27]. Enrichments and isolations proceeded in the manner described by Fries et al. [50] using VB broth and plates. All enrichments and reductions were performed aerobically at 10°C, with shaking (250 rpm). The isolates were labeled P2 (orange), P3 (off-white), and P4 (pale yellow), and the possible pair was labeled P1a&b (white colonies in the beginning and pink colonies developing over time). Preliminary Cr^6+ ^reduction observations of the three isolates in VB broth with 30 mg/l Cr^6+ ^revealed that only isolates P3 and P4 completely reduced 30 mg/l Cr^6+ ^in less than 60 days. Isolate P4 was observed to remove Cr^6+ ^faster than P3 and was consequently chosen for the isolate Cr^6+ ^reduction experiments. Isolation was confirmed via 16S identification as described below.

### Consortium and isolate reduction experiments

Both consortium and isolate P4 reduction experiments were conducted aerobically at 10°C, with shaking (250 rpm) and in triplicate. Consortium reductions consisted of 3 concentrations of Cr^6+ ^(0, 30, 60 mg/l final concentration) in 4.75 ml of VB broth. Final Cr^6+ ^concentrations (0, 30, 60 mg/l) were achieved using a 100× stock solution of Cr^6+ ^(3.735 g Cr^6+ ^in 10 ml distilled H_2_O). The inocula consisted of 250 μl of stationary phase enrichment culture (10^8^/ml) bringing the total volume for each tube to 5 ml.

Isolate reductions each consisted of 3 concentrations of Cr^6+^: 0, 25, 50 mg/l in 5 ml of VB broth. Cellular biomass was first established by growing isolate P4 in R_2 _broth (Bacto Yeast Extract, 0.5 g L^-1^; Bacto Proteose Peptone #3, 0.5 g L^-1^; Bacto Casamino Acids, 0.5 g L^-1^; Bacto Dextrose, 0.5 g L^-1^; Soluble Starch, 0.5 g L^-1^; Sodium Pyruvate, 0.3 g L^-1^; Dibasic Potassium Phosphate, 0.3 g L^-1^; Magnesium Sulfate, 0.05 g L^-1^) to stationary phase (OD = 0.15 at 600 nm 1:10 dilution in R2 broth). Cells were then centrifuged at 5,000 × g in a Sorvall microcentrifuge (Kendro Laboratory Products, Asheville, NC) and resuspended in VB broth.

Cell-free controls and Cr^6+ ^standards (0, 25, 50, 100 mg/l) were used as the baseline for detecting Cr^6+ ^reduction. Reduction was detected via the diphenyl carbazide assay (described below), measuring remaining Cr^6+^. All samples from the reduction cultures were assayed in triplicate, resulting in nine readings for each concentration. Averages and standard deviations were calculated using the spreadsheet program Excel (Microsoft) and graphed using CoPlot (CoHort Software V.6.2).

### Consortium Cr^6+ ^adsorption experiments

To confirm that observed decreases in Cr^6+ ^concentrations were due to reduction and not biosorption, three separate adsorption studies were conducted using killed cells (autoclaved), metabolically inhibited cells, and lyophilized cells. All cells were suspended in deionized water to limit metabolic activity. Live and killed (autoclaved) cells were obtained at stationary phase, centrifuged at 5,000 × g for 20 minutes, washed, centrifuged (Jouan, Thermo Electron Corp.) and resuspended in an equal amount of deionized water. Lyophilized cells (0.3 g) were suspended in 150 ml deionized water and allowed to rehydrate for one hour before use. Chromate concentrations were achieved using 100× stock solution of Cr^6+^.

Adsorption studies were conducted aerobically at 4, 10, 18 and 37°C, with shaking (250 rpm). Concentrations of Cr^6+^were analyzed by the diphenyl carbazide assay described below. Adsorption experiments were assayed in triplicate.

### Diphenyl carbazide assay

A diphenyl carbazide assay for measurement of Cr^6+ ^was developed from Standard Methods for the Examination of Water and Wastewater [51] as well as the methods listed in Turick *et al*. [52] with the following modifications. ChromaVer (diphenyl carbazide reagent) was obtained from Hach (Loveland, CO). Absorbance readings for reduction cultures and Cr^6+ ^standards of 0, 25, 50, 100 mg/l were recorded approximately every 48 hours for consortium cultures and every 12 hours for isolate cultures.

### DNA extractions

DNA was extracted from the isolate grown in VB broth. DNA extractions were performed using the Puregene DNA Isolation Kit (Gentra systems, Minneapolis, MN). Manufacturer's instructions for Gram Positive bacteria DNA extraction were used with the following changes: cells were pelleted by centrifuging at 16,000 × g for 5 minutes; lysis was performed at 90°C for 10 minutes; lysate was treated with RNase for 60 minutes; lysate was vortexed on low speed after protein precipitation solution was added and centrifuged at 16,000 × g for 5 minutes; DNA was precipitated with 100% isopropanol at -20°C overnight and then centrifuged at 16,000 × g for 5 minutes; DNA was then washed with ice cold 70% ethanol and centrifuged again at 16,000 × g for 5 minutes; ethanol was removed with a pipetter and the DNA was allowed to air dry at 37°C; DNA was re-hydrated by adding 100 μl molecular biology grade water and incubating overnight.

### PCR for 16S identification

PCR amplification for 16S identification was performed as described by Sheridan et.al. [53]. Fragments were then sent for sequencing on an ABI cycle sequencer at the Molecular Research Core Facility (Idaho State University, Pocatello, ID). Fragments were sequenced in both directions.

### Alignment and phylogenetics

The final contiguous sequence of 1373 base pairs was used to search both the Ribosomal Database Project  and Genbank  databases. Sequence fragments were aligned and analyzed as described in Sheridan et.al. [53]. The GenBank accession number for the 16S rRNA gene of isolate P4 is GenBank:DQ016989.

## Authors' contributions

WAA provided Hanford core samples and provided technical assistance. RNH performed the enrichments, isolations, reductions, adsorptions, DNA extraction, and PCR. VST lyophilized the consortium cells and provided technical assistance. RNH and PPS performed the alignment and phylogenetic analysis. RNH drafted the manuscript. All authors contributed to the experimental design and manuscript editing. All authors read and approved the final manuscript.
